# Ageing and dementia: age-period-cohort effects of policy intervention in England, 2006–2016

**DOI:** 10.1186/s12877-021-02341-4

**Published:** 2021-06-26

**Authors:** Kamila Kolpashnikova

**Affiliations:** University of Oxford, 42-43 Park End St, OX1 1JD Oxford, UK

**Keywords:** dementia, National Dementia Policy, Dementia Challenge, policy evaluation

## Abstract

**Background:**

Dementia is one of the most critical challenges of our time. According to the Dementia Statistics Hub, only about 66 % of all UK residents with dementia were diagnosed in 2017–2018. Yet, there are reservations about the early diagnosis of dementia-related diseases. As a result, the UK National Screening Committee does not recommend systematic population screening of dementia, although case-finding strategies are still applied for high-risk groups.

**Methods:**

This study added additional evidence of the effectiveness of the National Dementia Strategy and increased numbers of diagnosis of dementia on the younger cohorts of the older people, using the intrinsic estimator age-period-cohort (APC) models and the English Longitudinal Study of Ageing data.

**Results:**

Age effects show that diagnosis increases in volume only among those aged 75 and above, suggesting that many of those aged below 75 might not be diagnosed in time. Period effects show that although there was an initial increase due to the new policy implementation, the trend stalled in later years, indicating that the increase might not have been even across the period when controlled for age and cohort. The study also shows that cohort effects indicate lower prevalence in younger cohorts controlled for age and period effects.

**Conclusions:**

Although more research in diverse contexts is warranted, this study cautions against the abandonment of timely diagnosis, increased screening and case-finding, and shows some effectiveness of prevention strategies on the national level.

## Background

According to Dementia Statistics Hub, one in three people with dementia are left undiagnosed in the UK [1]. Between 2009 and 2015, the UK put tremendous efforts in addressing dementia in domestic health policy, including increasing diagnosis rates, promoting awareness and prevention, and changing treatment strategies [2]. This study attempts to contribute to the previous research and evaluate UK health policy effects on dementia diagnosis using the age-period-cohort (APC) analysis. It is particularly important to evaluate the effects of the national policy and strategy change, given the urge to evaluate its adequacy was expressed in recent work [3, 4] and the aftereffects of the COVID-19 pandemic on public health.

 The National Dementia Strategy (NDS) was launched in February 2009 to improve awareness about the illness, encourage an increase in diagnosis, prevention, and the quality of care for people diagnosed with dementia-related diseases. In 2012, NDS was superseded by Dementia Challenge, which was then updated to Dementia 2020 Challenge in 2015.

Among other changes with the UK-wide dementia policy, more people with mild dementia were prescribed acetylcholinesterase inhibitors, whereas antipsychotic prescriptions continued to decrease [2]. Donegan, Fox, Black, Livingston, Banerjee and Burns [2] report that the prescription of antidementia medication doubled in percentage in between 2005 and 2015 and reached 36.3 %, whereas the antipsychotic drug prescription halved from 22.1 to 11.4 %. Research shows that acetylcholinesterase inhibitors may slow down the cognitive decline in patients with Alzheimer’s disease, though more research is warranted [5, 6].

Although the present paper does not aim to call for the reversal of the current screening mandate in the UK, since the harmful effects of the early diagnosis need to be addressed and clear ethical and procedural measures outlined in the communication with the patients and caregivers [4], its objective is to provide new investigative evidence whether any changes were observed due to the implementation of the dementia-related national policy, particularly NDS and Dementia Challenge of 2012–2015. This aim is achieved by using improved methods in APC analysis, the intrinsic estimator (IE) models and the English Longitudinal Study of Ageing, 2006–2016. The IE models allow disentangling age, period, and cohort effects to illustrate whether the effects are constant or diverging over age groups, period, and cohorts.

As the age effects are expected to follow the national recommendations on screening and case-finding, this study is mostly interested in the analysis of period and cohort effects. The invariable period effects would suggest that there were no screening, case-finding, and diagnosis increase in the period, controlled for age and cohort, which we know is not factual in the case of the diagnosis of dementia in the analysed period [1]. On the one hand, the invariant cohort effects would confirm the assumption that prevention strategies, as well as the concurrent changes in treatment strategies, had no effect on the younger cohorts of people over 60 years of age. On the other hand, changes in the cohort trends might indicate some evidence of the opposite.

## Methods

### Data source

*Data.* The English Longitudinal Study of Ageing (ELSA) is used for the analysis [7] and was accessed through the UK Data Services.

*Study Design and Participants.* ELSA survey collection started in 2002 and interviewed people aged 50 years and older living in a private household in England. The original sample was based on the respondents who participated in the Health Survey for England in 1998, 1999, and 2001. Attrition levels in ELSA are, in general, higher than in comparable studies in the US (Health and Retirement Study), which can be explained by prior wave health conditions, passing away, lower literacy levels among attrited, and cultural differences in attitudes toward participating in longitudinal studies[8]. To deal with attrition, there were refreshment samples introduced to ELSA to keep the study’s representativeness. The survey waves for ELSA were scheduled for every other year, but the actual field collection spread over two years. That is why in the analysis, the actual years stretched from 2006 to 2017. This project uses Wave 3 (2006–2007) through 8 (2016–2017) to include a few years prior to the introduction of the NDS in 2009 and a few years after the Dementia Challenge of 2012–2015. The total ELSA sample in the selected waves was 59,807 people. After restricting the sample to those aged between 60 and 80, the analytical sample included 42,848 (72 % of the total sample) people. The analytical model (the intrinsic estimator model, explained below) requires data of a certain format, where the data should be pooled into a rectangular age-by-period array (such as represented in the ‘Dementia by Age Group (%)’ part of Table 1). Thus, cohorts above age 84 in the periods between 2010 and 2017 had to be disregarded in the analysis (see Table 1). There were no missing values because this study used the birth year (present for all waves) and the year of interview (present for all waves) variables to analyse APC effects.

### Outcome variable: prevalence of dementia-related disease

The dependent variable is measured by whether the diagnoses of dementia-related diseases, including Alzheimer’s disease, were reported at the surveyed wave. The outcome is a dummy variable, 1 for ‘yes’ and 0 for ‘no’.

Table 1 summarizes the descriptive statistics for the dementia-related disease prevalence rates by age group and cohorts. It shows that the prevalence rate increases with age and that it is higher in older cohorts, as expected. The table also shows that the increased diagnosis in the pre-2015 period reported more cases of dementia for younger older people (in between 65 and 74) and among those who were 80–84 years of age.

**Table 1:** Prevalence rates by age and period and cohort and period, in %

	Period			
	2006–2009	2010–2014	2015–2017	Total
Dementia by Age Group (%)				
60–64	0.61	0.41	0.43	0.49
65–69	0.50	0.74	0.61	0.65
70–74	0.51	1.15	0.63	0.84
75–79	1.18	1.74	2.37	1.70
80–84	1.83	4.75	3.79	3.65
Dementia by Cohorts (%)				
1925–1929	1.83	0	0	1.83
1930–1934	1.18	4.75	0	3.10
1935–1939	0.51	1.74	3.79	1.62
1940–1944	0.50	1.15	2.37	1.13
1945–1949	0.61	0.74	0.63	0.68
1950–1954	0	0.41	0.61	0.47
1955–1959	0	0	0.43	0.43
Total	13,346	21,304	8198	42,848

### Age, period, and cohort variables

The analysis is based on the five-year intervals of the categories of age, period, and cohort, which are usually used in the analysis of APC effects [9–11]. The intervals of five years were used in the construction of age and cohort categories. However, the three resulting period categories were constrained by the actual years of the survey, 2006–2017. Thus, the resulting period categories were 2006–2009, 2010–2014, and 2015–2017.

### Analytical strategy

This paper’s models employ the intrinsic estimator (IE) to disentangle APC effects in dementia-related diseases [9, 12]. The IE modelling in APC analysis remains the most appropriate way to analyse the effects without having to impose constraints on either age, period, or cohort categories [10, 11, 13]. Some critiques of the method exist. For instance, L Luo [14] showed that IE models would not work in all situations, using simulations. However, later, RK Masters, DA Powers, RA Hummer, A Beck, S-F Lin and BK Finch [13] showed that the situations where IE models will not work are very unlikely to happen in the real world and reclaimed the confidence in IE models in APC analysis.

In public health research, Bell’s HAPC (hierarchical age-period-cohort) models, developed in A Bell [15], are used more commonly than the IE models. However, the HAPC models require strong assumptions regarding (usually) period effects [16]. Considering that period effects are expected to vary between 2006 and 2017 in England’s dementia prevalence trends, IE models are preferred in the present paper. We did not include independent variables because the results for APC effects in IE models do not depend on independent variables or weights [12].

## Results

Table 2 presents the results from the IE models on dementia-related diseases’ prevalence. Most APC effects are significant in the model. Model 1 shows that the prevalence increases with age and period and decreases with a cohort change, except for the cohort born during WWII and the oldest cohort.

**Table 2:** Intrinsic estimator age-period-cohort effects models on dementia-related diseases prevalence

	Coefficients	SE	Confidence Interval
Age Group			
60–64	-0.403^***^	(0.119)	[-0.635, -0.170]
65–69	-0.535^***^	(0.119)	[-0.767, -0.303]
70–74	-0.477^***^	(0.121)	[-0.714, -0.239]
75–79	0.286^**^	(0.093)	[0.103, 0.469]
80–84	1.128^***^	(0.092)	[0.948, 1.309]
Period			
2006–2009	-0.347^***^	(0.078)	[-0.500, -0.194]
2010–2014	0.172^*^	(0.067)	[0.040, 0.304]
2015–2017	0.175^*^	(0.081)	[0.017, 0.333]
Cohorts			
1925–1929	-0.102	(0.144)	[-0.384, 0.179]
1930–1934	0.350^**^	(0.118)	[0.118, 0.581]
1935–1939	0.157	(0.119)	[-0.076, 0.390]
1940–1944	0.458^**^	(0.143)	[0.177, 0.739]
1945–1949	0.138	(0.137)	[-0.130, 0.406]
1950–1954	-0.434^**^	(0.145)	[-0.717, -0.150]
1955–1959	-0.567	(0.354)	[-1.261, 0.127]
Constant	-4.661^***^	(0.080)	[-4.818, -4.504]
Observations	42,848		
Deviance	5211.749		
Log-Lik.	-2605.875		

Standard errors in parentheses. ^+^*p* < 0.10, ^*^p < 0.05, ^**^p < 0.01, ^***^p < 0.001

## Discussions

### Age effects

**Fig. 1:**
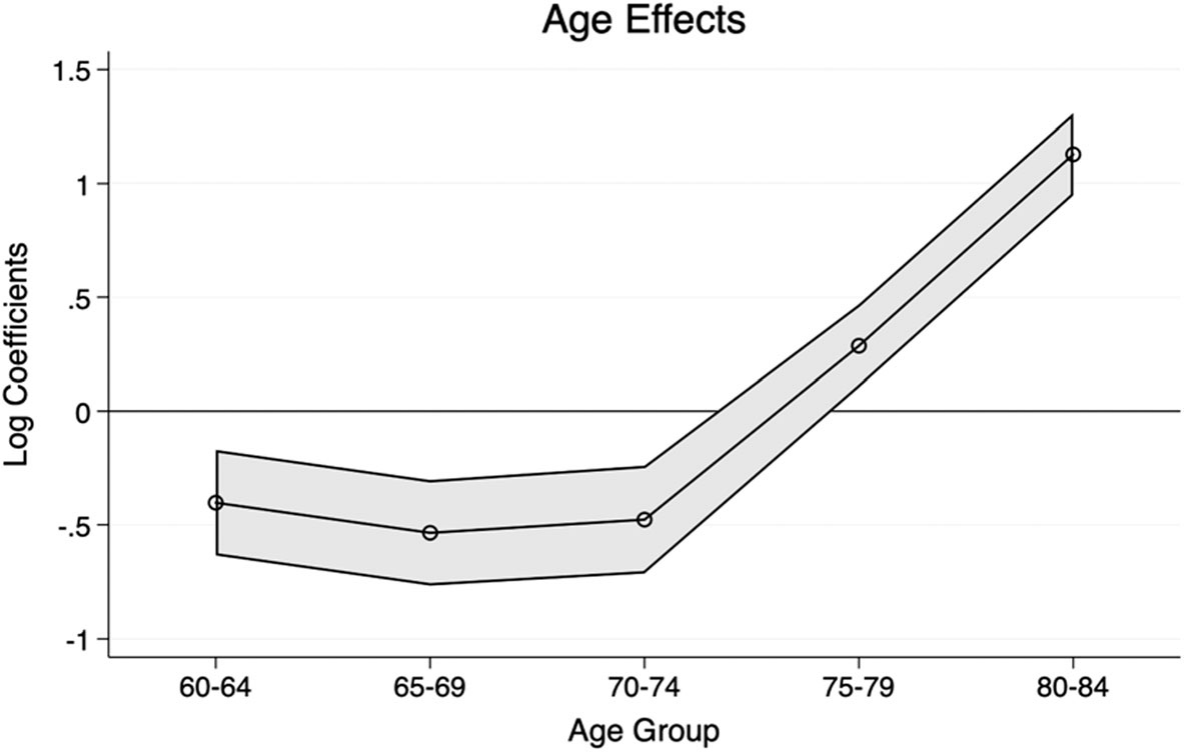
Age effects

Age effects based on the IE model in Table 2 are presented in Fig. 1. The trends reveal that the diagnosis (preceded by screening and case-finding) mostly happen among people aged 75 to 84. This indicates that even during the analysed period since the start of the NDS with a policy mandate for increased testing of dementia, those aged 74 and below were rarely screened and diagnosed with dementia. The descriptive findings in Table 1 confirm this result for people of age 69 and below. For those between 70 and 74, the prevalence rate doubled between the first and the second period and reverted to the first period’s levels in the third period. These results suggest that very few younger older people are diagnosed in a timely manner, even after the NDS, reflecting the current recommendations of the UK National Screening Committee. The steep increase for those aged 75 and above on the age effects confirms that many of these cases might remain undetected earlier if we assume that the prevalence increases gradually rather than exponentially over age groups. The increase also confirms that diagnosis is more prevalent in older cohorts. These results of the age effects are expected and are according to the national guidelines on the screening, case-finding, and diagnosis of dementia-related diseases.

### Period effects

**Fig. 2:**
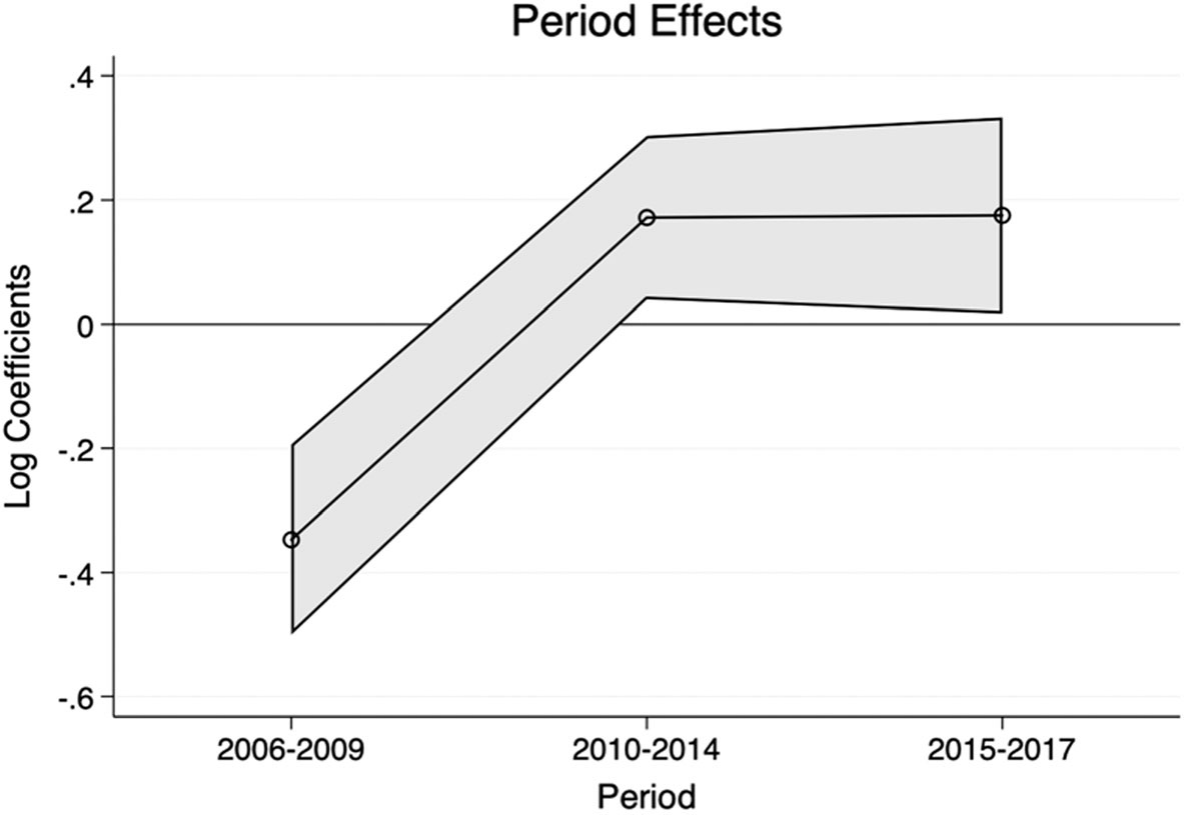
Period effects

Period effects, presented in Fig. 2, confirm that the period after 2009 since the inception of NDS was associated with increased dementia diagnosis (prevalence) in the population. The period effects, however, stalled since the recommended screening remained only for those over 75. The trends show a steep increase between the first period (2006–2009), followed by stagnation between 2010 and 2014 and 2015–2017 when controlled for age and cohort effects. Overall, the period effects confirm that the call for increased diagnosis in dementia was delivered since the implementation of the NDS but stalled in recent years.

### Cohort effects

**Fig. 3:**
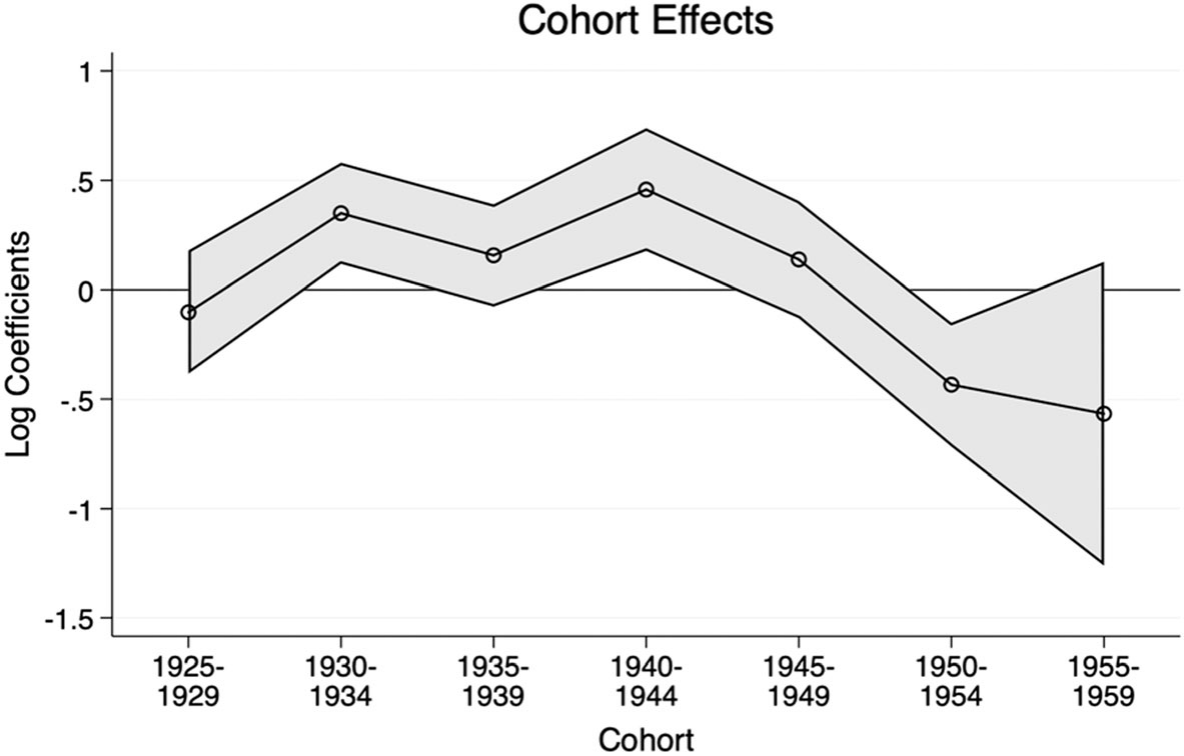
Cohort effects

The cohort effects in this study can potentially show the effectiveness of the dementia risk prevention policy if a decreasing trend in prevalence is detected in earlier cohorts, net of age and period effects (see Fig. 3). The trends show that the risk prevention activities, treatment strategy changes, as well as changes in the lifestyle of the younger cohorts not related to the policy implementation might have positively affected the younger cohorts, particularly those born between 1950 and 1954. Moreover, the cohort of those born in 1950–1954 is the first cohort who would have been part of the National Health Service from birth, unlike the earlier cohorts. Striking differences in prevalence can be confirmed between the younger cohort in the survey and those who are ten years older than them, which gives us a bit of optimism in terms of the effects of the risk prevention measures, although the association might be explained by other factors unrelated to the policy implementation as well. More extended observation is warranted to be able to say anything definitively.

## Conclusions

This study provides additional evidence of the effectiveness of the UK National Dementia Strategy and Dementia Challenge of 2012–2015, particularly of the timely diagnosis of dementia and risk prevention, based on the intrinsic estimator APC analysis. The study provides a novel way to use APC models to analyse the effectiveness of preventive and diagnostic measures using longitudinal data.

The findings confirm that age effects follow the screening recommendations on the national level. The period effects reveal a substantial increase in diagnosis following the implementation of the National Dementia Strategy and consequent stall in the trend in the later years. The cohort effects show some improvement in younger cohorts, suggesting the effectiveness of preventive policy or generational changes in lifestyles.

Nonetheless, the evidence presented in this study is not conclusive because of the limited data, and more research is warranted, particularly using a range of data from diverse contexts. The way to do this is to analyse the APC effects across different contexts, depending on their dementia-detection strategies and selected preventive measures, including in other nations of the UK (Scotland, Wales, and North Ireland), as well as European, North American, Asian, and other contexts. The analysis within different nations of the UK is important because of the differences in approaches to diagnoses and diagnostic support across the four nations, which closely intertwined with patients’ decision-making on usefulness of a diagnosis and ultimately on whether the diagnosis is sought out.

One limitation of this study is that the prevalence of early detection of dementia-related diseases in the younger cohorts can also be related to other cohort-related changes: lifestyles and decreases in alcohol consumption and smoking, among other things. The policy push toward controlling and decreasing smoking among the population in the late 90 s-early 2000 s has had a tremendous effect on the perception of public smoking and second-hand smoking, which affected the numbers of smokers across many nations. Already preceding England’s smoking ban in the workplace in 2007, the public opinion toward smoking has shifted dramatically.

Another limitation is the high rates of attrition in ELSA. In this study, this is particularly relating to the mortality rates in older cohorts. The use of IE models, which require a rectangular shape of the data, helps the analysis because the older cohorts are removed from the analysis. Still, the results of this study regarding the older cohorts (particularly of those born between 1925 and 1929) might be biased because healthier older people live longer years, whereas the mortality rate is higher among older people with dementia-related conditions. The interpretations of the present study’s results regarding older cohorts must take into consideration that real prevalence rates would be higher.

Despite the limitations of this study, the data used are from the national survey, which makes the results of this study generalisable to older people living in England with some caution as in the limitations discussed above.

Future research could focus on the effects of preventive measures and increased diagnosis over longer periods of time, particularly focusing on case-finding among high-risk populations and other dementia detection strategies.

## Data Availability

The datasets generated and/or analysed during the current study are available in the UK Data Service, https://beta.ukdataservice.ac.uk/datacatalogue/series/series?id=200011.

## Abbreviations

APC: age-period-cohort
ELSA: English Longitudinal Study of Ageing
HAPC: hierarchical age-period-cohort
IE: intrinsic estimator
NDS: National Dementia Strategy
